# The penetration effect of HMME-mediated low-frequency and low-intensity ultrasound against the *Staphylococcus aureus* bacterial biofilm

**DOI:** 10.1186/s40001-020-00452-z

**Published:** 2020-10-22

**Authors:** Tao Wang, Wei Ma, Zhinan Jiang, Liangjia Bi

**Affiliations:** 1 Department of Stomatology, The Fourth Affiliated Hospital, Harbin Medical University, YinHang Street, Nangan District, P.O. Box 31, Harbin, 150001 China; 2 Department of Periodontics, WuHan First Stomatological Hospital, WuHan, 430000 China

**Keywords:** Low-frequency and low-intensity, Sinusoidal ultrasound, *Staphylococcus aureus*, Biofilm, Permeability

## Abstract

**Background:**

The purpose of this study was to observe the effect of hematoporphyrin monomethyl ether (HMME)-mediated low-frequency and low-intensity ultrasound on mature and stable *Staphylococcus aureus *(*S. aureus*) biofilms under different ultrasound parameters.

**Methods:**

The biofilm was formed after 48-h culture with stable concentration of bacterial solution. Different types of ultrasound and time were applied to the biofilm, and the ultrasonic type and time of our experiments were determined when the biofilm was not damaged. The penetration effects of low-frequency and low-intensity ultrasound were decided by the amount of HMME that penetrated into the biofilm which was determined by fluorescence spectrometry.

**Results:**

The destruction of biofilms by pulse waveform was the strongest. Sinusoidal low-frequency and low-intensity ultrasound can enhance the biofilm permeability. For a period of time after the ultrasound was applied, the biofilm permeability increased, however, changes faded away over time.

**Conclusions:**

Low-frequency and low-intensity sinusoidal ultrasound significantly increased the permeability of the biofilms, which was positively correlated with the time and the intensity of ultrasound. Simultaneous action of ultrasound and HMME was the most effective way to increase the permeability of the biofilms.

## Background

Bacterial biofilm is a microcolony aggregation formed by a single or multiple bacterium to adapt to the natural environment. Biofilms are mainly composed of biofilm substrates, bacterial cells and water-borne channels distributed in biofilm substrates. The main component of biofilm is extracellular polymeric substances (EPS) [1, 2]. The main role of EPS is to protect bacterial cell in the biofilm from dehydration in adverse environment, and to defend immune substances produced by the body and exogenous antimicrobial drugs [3, 4]. Water-borne channels are the main pathway to transport nutrients and oxygen, which can enhance the adaptability of bacteria in biofilm [5]. A morphological change is when bacteria adapt to environmental changes, it enhances the resistance of bacteria to external environment. So, many refractory infections are related to the formation of biofilms.

Because of the characteristics of bacterial biofilms, the treatment for them will face many difficulties. At present, the main methods used to remove bacterial biofilms include mechanical methods and antibacterial drug therapy, but they are not satisfactory [6]. Because there are some shortcomings, and cannot achieve very satisfactory results. Mechanical removal relies on the patient's high compliance and surgeon's skills. Only when these two factors are satisfied better results can be achieved [7]. Long-term drug treatment can cause drug resistance and dysbacteriosis [8], because of the protective effect of biofilm, the effect of antibiotics is poor. We hope to avoid these problems clinically, therefore, how to find ways to destroy the protective effect of biofilm has become a research hotspot.

The effect of ultrasound on biofilm is related to the intensity of ultrasound [9, 10]. EPS can be destroyed by high-intensity ultrasound, resulting in sterilization. However, low-frequency and low-intensity ultrasound are beneficial to the maturation of biofilms [11, 12]. The reason of these two phenomena is related to the effect of ultrasound on biofilm structure. Firstly, cavitation has a destructive effect on extracellular substances. Secondly, in the depth of biofilm, the energy of ultrasound is weak, but it plays a big role in promoting the transport of nutrient oxygen. Which of the two will be the dominant one to determine the effect of ultrasound. Therefore, low-frequency and low-intensity ultrasound can only kill bacteria when combined with antibiotics. The reason may be that ultrasound enhances the drug's ability to penetrate the biofilm. Ultrasound also speeds up the metabolism of bacteria in biofilms that increases the intake of antibiotic drugs [13]. In conclusion, high-intensity ultrasound has a strong destructive effect on biofilm and low-intensity ultrasound combined with antibiotics can achieve a relatively significant synergistic sterilization effect [14].

**Fig. 1:**
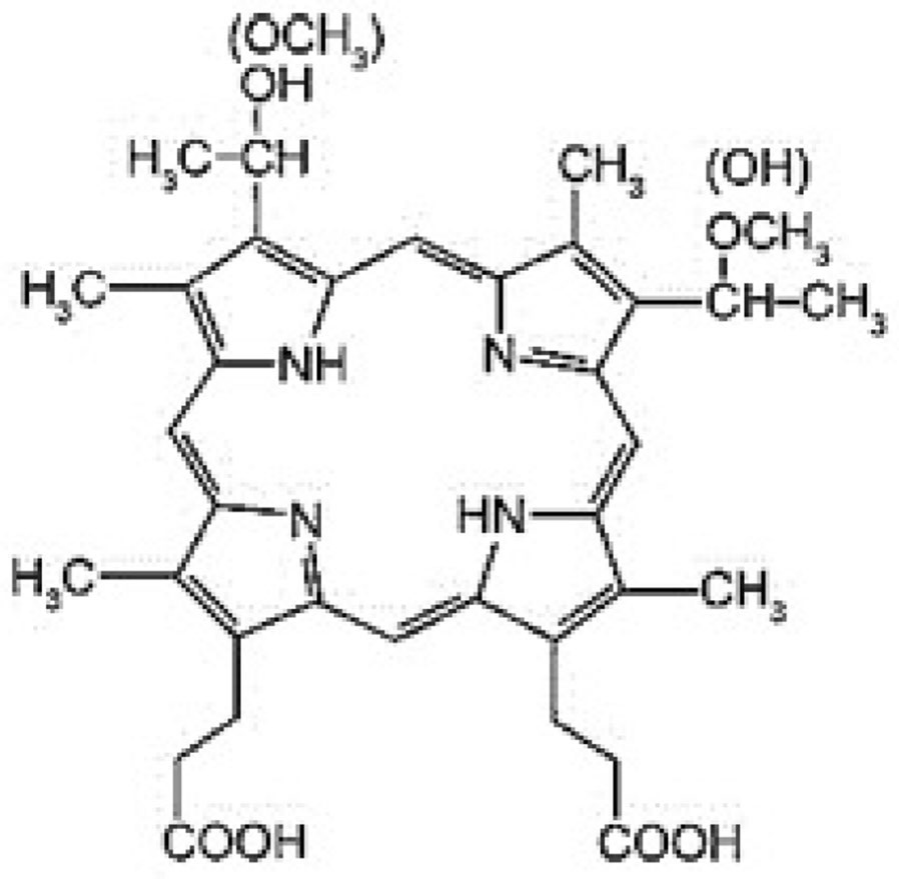
The chemical structure of HMME

3-(1-Methoxyethyl)-8-(1-hydroxyethyl)-porphyrin IX or 8-(1-methoxyethyl)-3-(1-hydroxyethyl)-porphyrin IX is the chemical name of hematoporphyrin monomethyl ether (HMME), and they are also isomers of each other. Its chemical structure is shown in Fig. 1 [15]. Approved by the Unite States Food and Drug Administration (FDA), HMME is the only legitimate clinical application and it has been proved safe and effective by experiments. Compared with the first-generation of hematoporphyrin derivative (HpD), the toxicity of HMME to human body is very low in both short and long term, higher ROS (reactive oxygen species) mass yield, stronger photodynamic effect, faster clearance with surrounding normal tissue, etc. In addition, HMME has a stronger characteristic in fluorescence spectrum. The content of HMME can be known by measuring its fluorescence spectrum.

At present, PDT (photodynamic therapy) and SDT (sonodynamic therapy) have shown a bactericidal effect, but the current research is mostly limited to plankton bacteria [16, 17]. Research on the treatment of bacterial biofilms are still rare. This study focused on the biological effects and osmotic effect of low-frequency and low-intensity ultrasound on bacterial biofilm model. We attempt to provide an experimental basis and an idea for the effect of therapeutic ultrasound on bacterial biofilm.

## Methods

### Bacteria and culture conditions

*S. aureus* (ATCC 6538) was cultured in Luria–Bertani (LB) broth at 37 °C for 48 h. The bacteria were washed three times with sterile saline and the optical density (OD 630 nm) set to a level of 0.2 (about 10^8^ CFU mL − 1). To be specific, after centrifugation of the bacterial suspension, the upper culture solution was poured out, and then sterile saline was poured in. After mixing, the solution was centrifuged again and the supernatant was poured out, which was repeated three times, in order to make the OD value more accurate and eliminate the interference of the culture solution.

### Experimental drug

HMME (ampoule, 5 ml/piece, 5 mg/ml) was provided by the Laboratory for Antimalarial Drug Research of the Second Military Medical University (Shanghai, China). The HMME was diluted twice with sterile saline so that the storage concentration was 10 mg/ml and kept in darkness at − 20 °C.

One of the reasons why HMME is selected in this experiment is that it has fluorescence, which is convenient for fluorescence spectrum analysis. Another reason is that it is a photoacoustic sensitizer, which has bactericidal effect when irradiated by laser or ultrasound.

### Ultrasonic generator

The ultrasonic generator in our experiment was designed and manufactured by Harbin Institute of Technology (Harbin, China. frequency: 25 kHz, ultrasonic intensity: 0.5–3 W/cm^2^, duty cycle: 50%). The ultrasonic device in this experiment contained sine wave and pulse wave. The specific parameters of the ultrasonic instrument were measured and calibrated by hydrophone under the same experimental conditions. The sound intensity had three adjustable gears of 0.5, 1 and 1.5 W/cm^2^.

### Culture of bacterial biofilm

Determination of the optical density (OD) value of the bacterial suspensions: the bacterial density in the suspensions can be roughly estimated by measuring the OD value, but calibration is required before use. The detailed steps are as follows:

Samples were divided into the following 18 time points: 0.5, 1, 2, 2.5, 3, 3.5, 4, 5, 6, 7, 8, 10, 12, 14, 16, 18, 20, 22 h, respectively. OD 630 value of bacterial suspension at each point.

Prepare 19*3 = 57 broth solutions. Fill 1 mL broth solution into 2 mL EP (Eppendorf) tube. Inoculate the 57 broth solutions with the same concentration of bacterial solution of 100 L and shake well.

After inoculation, 54 strains were incubated in the same incubator at the same time. Three of them were used for spectrophotometer calibration and repeated three times.

At each scheduled time point, 3 strains of bacteria solution were taken from the incubator. Each sample was measured for 3 times, and the average value was taken as the OD 630 value of the sample.

The growth curve of *S. aureus* was plotted and the bacterial solution in a stable concentration period was applied to inoculate the bacterial biofilm. The previously sterilized microporous filter membrane was spread in a 3-grid LB agar plate, and one filter membrane was laid in each grid of the plate. 50 μL purified bacterial was then uniformly coated on the filter membrane at 37 °C. After 48 h of cultivation, tight and thin *S. aureus* biofilm was formed.

### The damage degree of ultrasound on biofilm

Safe parameters and time were determined by observing the damage degree of samples under different ultrasonic parameters and time. The experiment was randomly divided into three groups (A, B, C) (three samples per group), as shown in Table 1.

**Table 1:** The group setup

	Group 1			Group 2			Group 3		
Saline volume (mL)	1			1			1		
Incubation time (min)	1	2	3	3			3		
Ultrasound	–			Sinusoidal			Pulsed		
Intensity (W/cm^2^)	–			0.5	1.0	1.5	0.5	1.0	1.5
Reagent volume (mL)	1			1			1		
Reaction time (min)	1			1			1		

Group A was blank group, which was designed to use saline (1 mL) for different incubation times (1, 2, 3 min), Group B was sinusoidal ultrasound group. Each sample was incubated with 1 mL normal saline for 3 min, and additional sinusoidal ultrasound (1. 0.5 W/cm^2^, 2. 1  W/cm^2^, 3. 1.5 W/cm^2^) and Group C was pulsed ultrasound group. Each sample was incubated with 1 mL normal saline for 3 min, and additional pulsed ultrasound (1. 0.5 W/cm^2^, 2. 1 W/cm^2^, 3. 1.5 W/cm^2^). After all the steps above, 1 mL of plaque display reagent was added to all groups of samples and incubated for about 1 min. Then all samples were washed with normal saline for 3 times.

### Spectral curves of HMME solutions with different concentrations

**Fig. 2:**
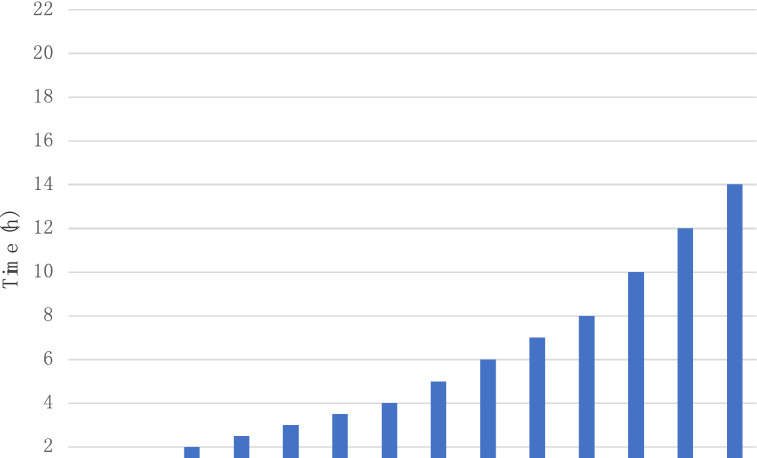
The setup of cultivating time

**Fig. 3:**
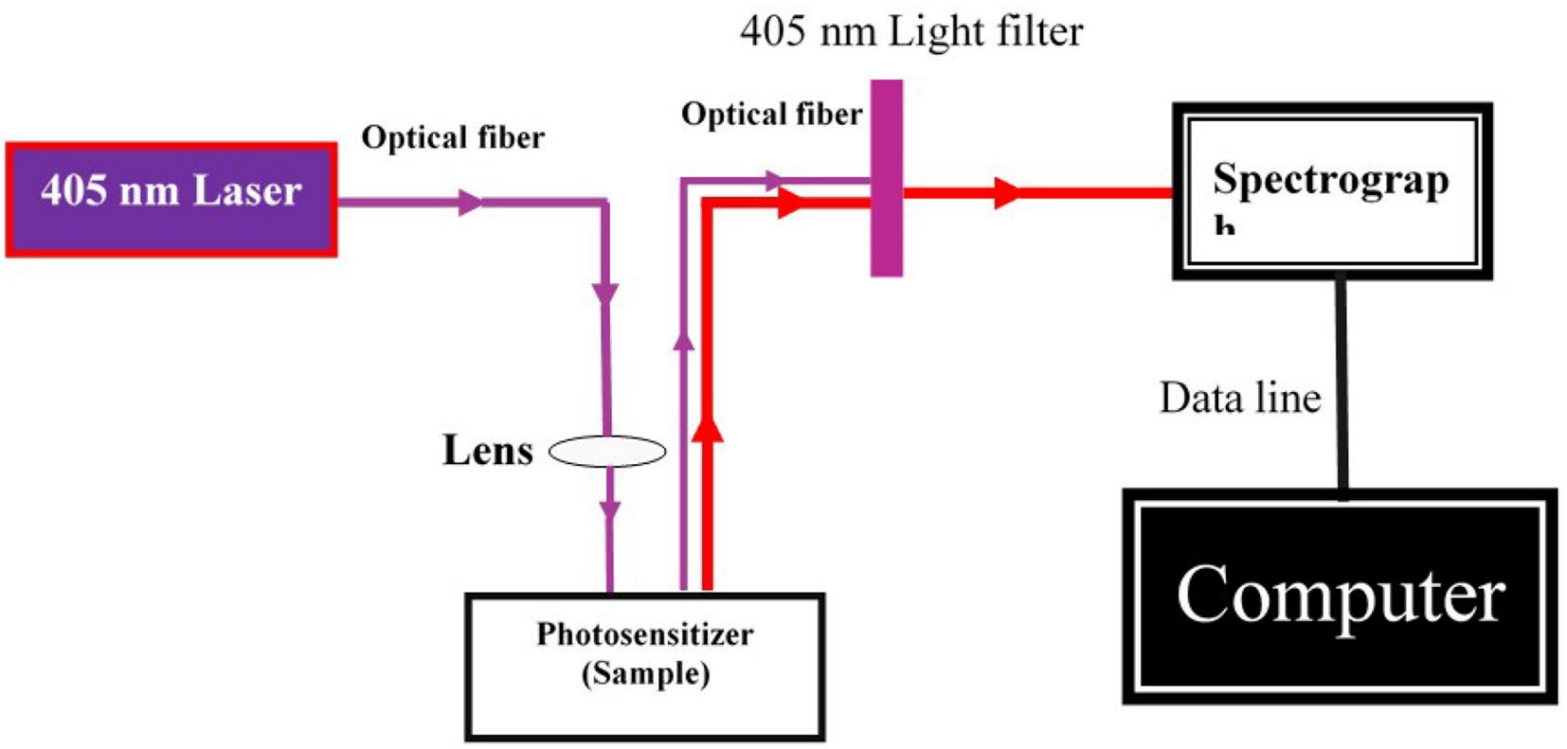
The mode of fluorescence spectrometric pattern

The 5 mg/mL HMME was gradient diluted into solutions of 1, 2, 4, 6, 10, 15, and 30 μg/mL, respectively. The model of the fluorescence spectrometry system is shown in Fig. 2. Diode laser emitter with wavelength 405 nm (HPD7404, USA) was the laser light source, as shown in Fig. 3. The HMME solution of each concentration was determined by spectrometry three times and the integral area under the spectral curve was calculated.

### Low-frequency and low-intensity ultrasonic penetration

To study the penetration effects of low-frequency and low-intensity ultrasound, each biofilm sample was put into a 35-mm plate and randomly divided into four groups (1–4): Group 1 was control group. Incubation was carried out with 1 mL normal saline alone (0.5, 1, 1.5, 2, 2.5 and 3 min) and intensity of sinusoidal ultrasound was: 0, 0.5 and 1 W/cm^2^. Group 2 was single-drug group. Incubation was carried out with 1 mL 25 g/mL HMME alone (0.5, 1, 1.5, 2, 2.5 and 3 min) and no ultrasound was added. Group 3 was the HMME added after ultrasound treatment (ultrasound treatment before). Incubation was carried out with 1 mL normal saline alone (0.5, 1, 1.5, 2, 2.5 and 3 min) and intensity of sinusoidal ultrasound was, respectively, 0.5 and 1 W/cm^2^. Then 25 μg/mL HMME solution (1 mL) was added to incubation. Fluorescence spectra were measured at the corresponding time points of 0.5, 1, 1.5, 2, 2.5 and 3 min after incubation. Group 4 was the ultrasound treatment and HMME were administered simultaneously (simultaneously). 25 μg/mL HMME was added to each sample for incubation, sinusoidal ultrasound (0.5 and 1 W/cm^2^) was added, respectively. Fluorescence spectra were measured at the corresponding time points of 0.5, 1, 1.5, 2, 2.5 and 3 min after incubation.

### Statistical analysis

The PC program SPSS 22.0 software was used for statistical analysis. The data were expressed as the mean ± standard deviation. The differences between the two sinusoidal ultrasound (0.5 and 1 W/cm^2^) at same period were statistically analyzed by the univariate ANOVA. Differences were considered to be significant when *P* < 0.05.

## Results

### The growth curve of *S. aureus*

**Fig. 4:**
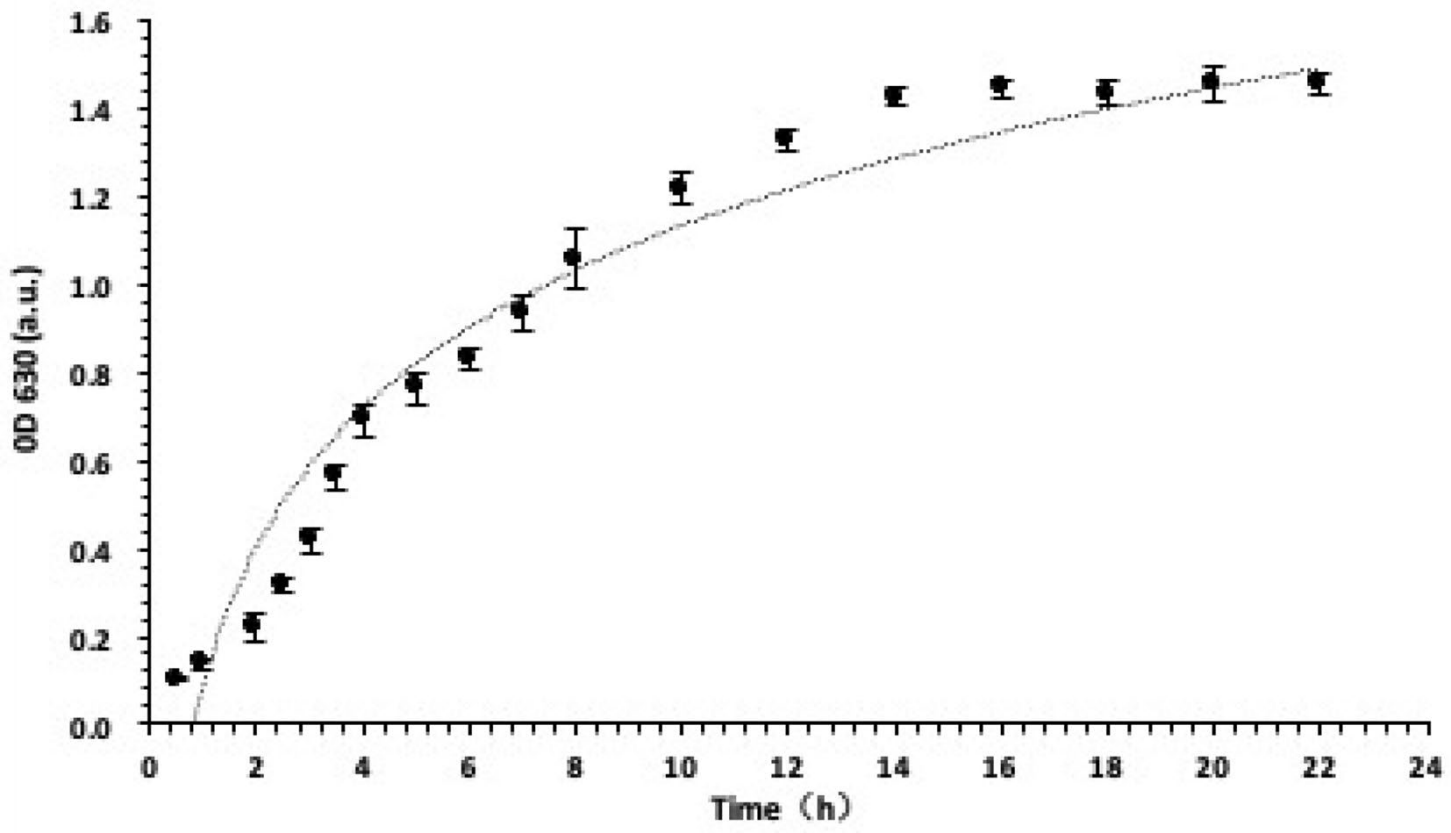
Evaluation of *S. aureus* growth in LB medium. OD 630 value of bacteria in LB medium at each time point is marked as a filled circle in the figure. Data represent mean values (*n* = 10), and error bars represent standard deviations.

The growth curve of *S. aureus* was plotted based on the OD 630 value at each time point repeatedly to evaluate the growth of *S. aureus* in LB medium. Figure 4 shows that *S. aureus* did not immediately start rapid proliferation after inoculation. After about 2 h, it began to enter the logarithmic growth phase. The OD 630 value start to slow down 14 h after inoculation, indicating that the growth rate of bacteria began to decrease, beginning to enter a period of stability. So, using the bacteria of this period can guarantee the stability of biofilm.

### The damage degree of ultrasound on biofilm

**Fig. 5:**
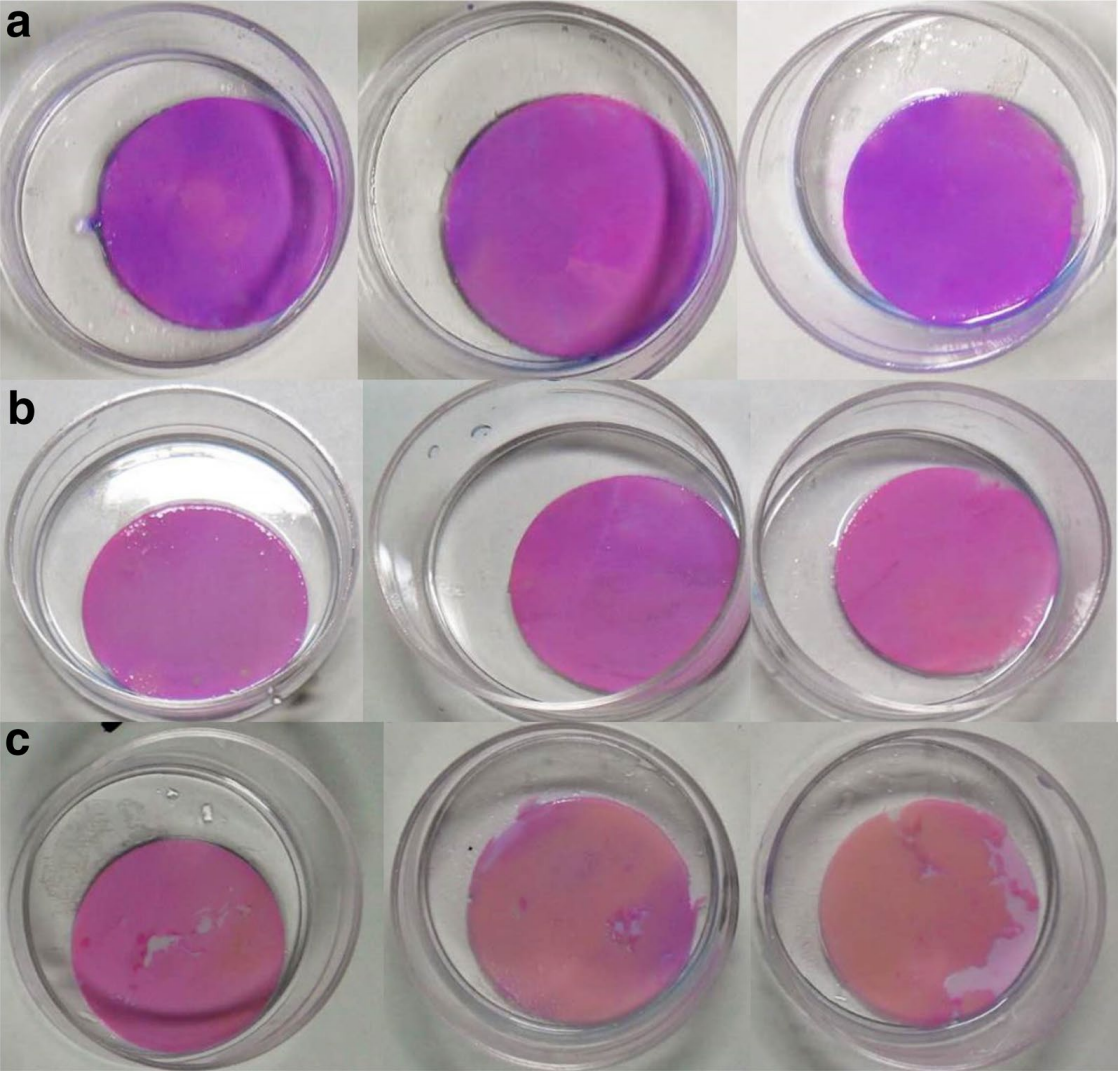
The comparison of the destruction degree of samples under different treatment conditions. **a** is the control group. From left to right, only 1 mL saline is incubated for 1.2.3 min, respectively. **B** is the sinusoidal ultrasound group. From left to right, 1 mL saline is incubated for 3 min and the intensity of sinusoidal ultrasound is 0.5, 1 and 1.5 W/cm^2^, respectively. **c** is the pulse ultrasound group. From left to right, 1 ml saline is incubated for 3 min and the intensity of pulse ultrasound is 0.5, 1 and 1.5 W/cm^2^, respectively.

After all samples were stained with the plaque indicator, the damage degree of samples under various treatment conditions was observed with the naked eye. The results are shown in Fig. 5. Figure 5a is the blank control group. From left to right, 1 ml saline is incubated for 1, 2, 3 min, respectively. After staining, no obvious damage marks were found, and there was no significant difference among the samples. It shows that saline has no obvious destructive effect on biofilm. Figure 5b is the sinusoidal ultrasound group. From left to right, 1 ml saline is incubated for 3 min and the intensity of sinusoidal ultrasound is 0.5^2^, 1 and 1.5 W/cm^2^, respectively. Only when the ultrasound intensity is 1.5 W/cm^2^ does the biofilm damage occur. While 0.5 and 1 W/cm^2^ ultrasound do not cause obvious damage to the biofilm samples.

Therefore, 0.5 and 1 W/cm^2^ sinusoidal ultrasonic intensity are selected in the relevant penetration promotion experiment. Figure 5c is the pulse ultrasound group. From left to right, 1 ml saline is incubated for 3 min and the intensity of pulse ultrasound is 0.5, 1 and 1.5 W/cm^2^, respectively. All 3 ultrasound intensities cause varying degrees of damage to biofilm samples. This indicates that the ultrasonic pulse waveform has a stronger destructive effect on biofilm samples. It is not suitable to study the penetration effect of low-frequency and low-intensity ultrasound on biofilm.

### Spectral curves of HMME solutions with different concentrations

**Fig. 6:**
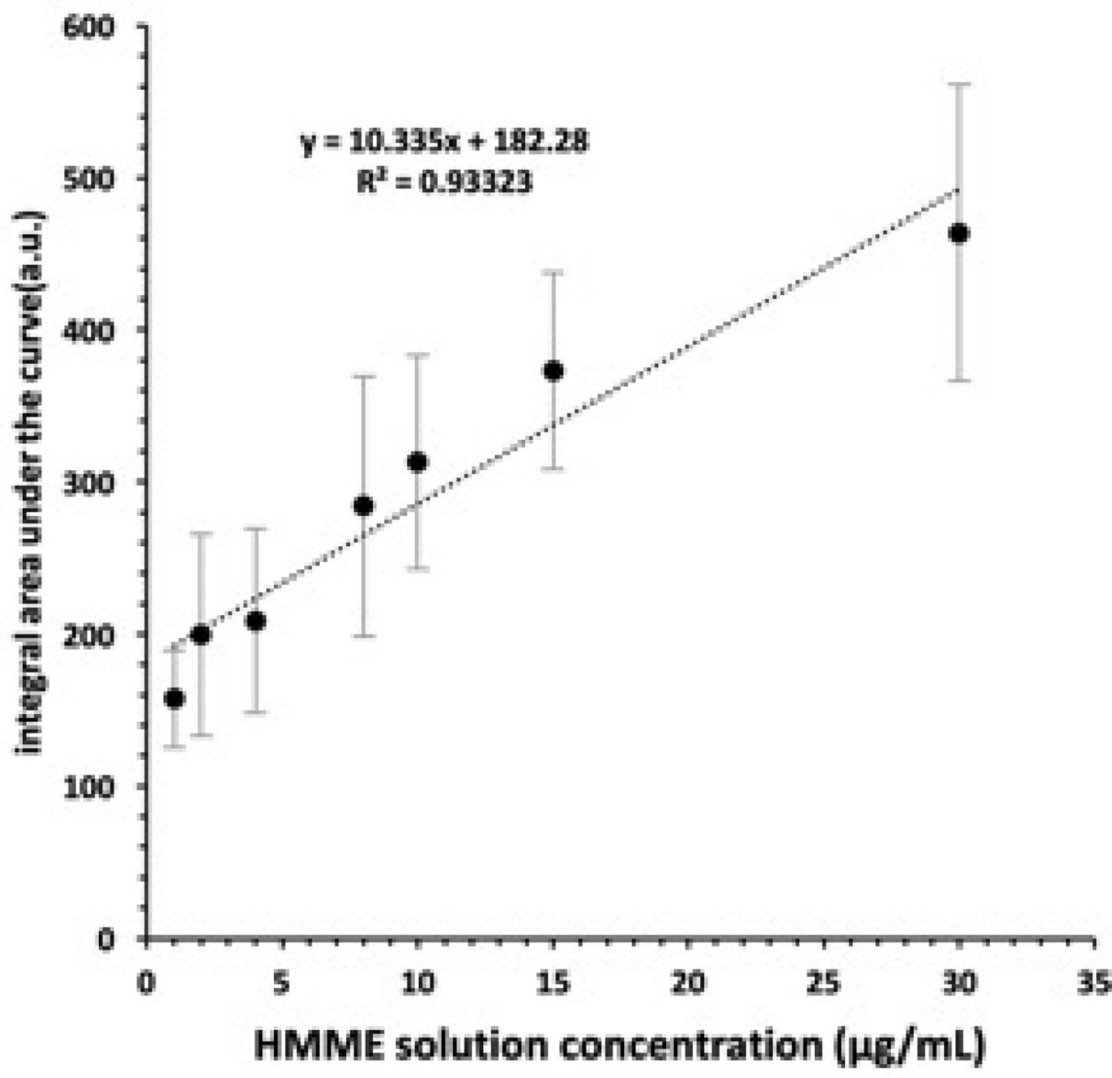
The correlation between the integral area under HMME fluorescence spectrum curve and concentration (1, 2, 4, 6, 10, 15, and 30 μg/mL HMME). Data represent mean values (*n* = 3), and error bars represent standard deviations

Fluorescence spectra of 1, 2, 4, 6, 10, 15, 30 μg/mL HMME were determined. The integral area under each curve S was measured. Linear fitting was made between the S of each sample and the corresponding solution concentration C. S represented the integral area under the fluorescence spectrum curve, and C represented the concentration of HMME. As shown in Fig. 6, the fitting equation can be obtained: S = 10.335x + 182.28. Accordingly, the S value could be used to reflect the amount of HMME. In other words, the higher S value was, the higher HMME amount was, and vice versa.

### Low-frequency and low-intensity ultrasonic penetration

**Fig. 7:**
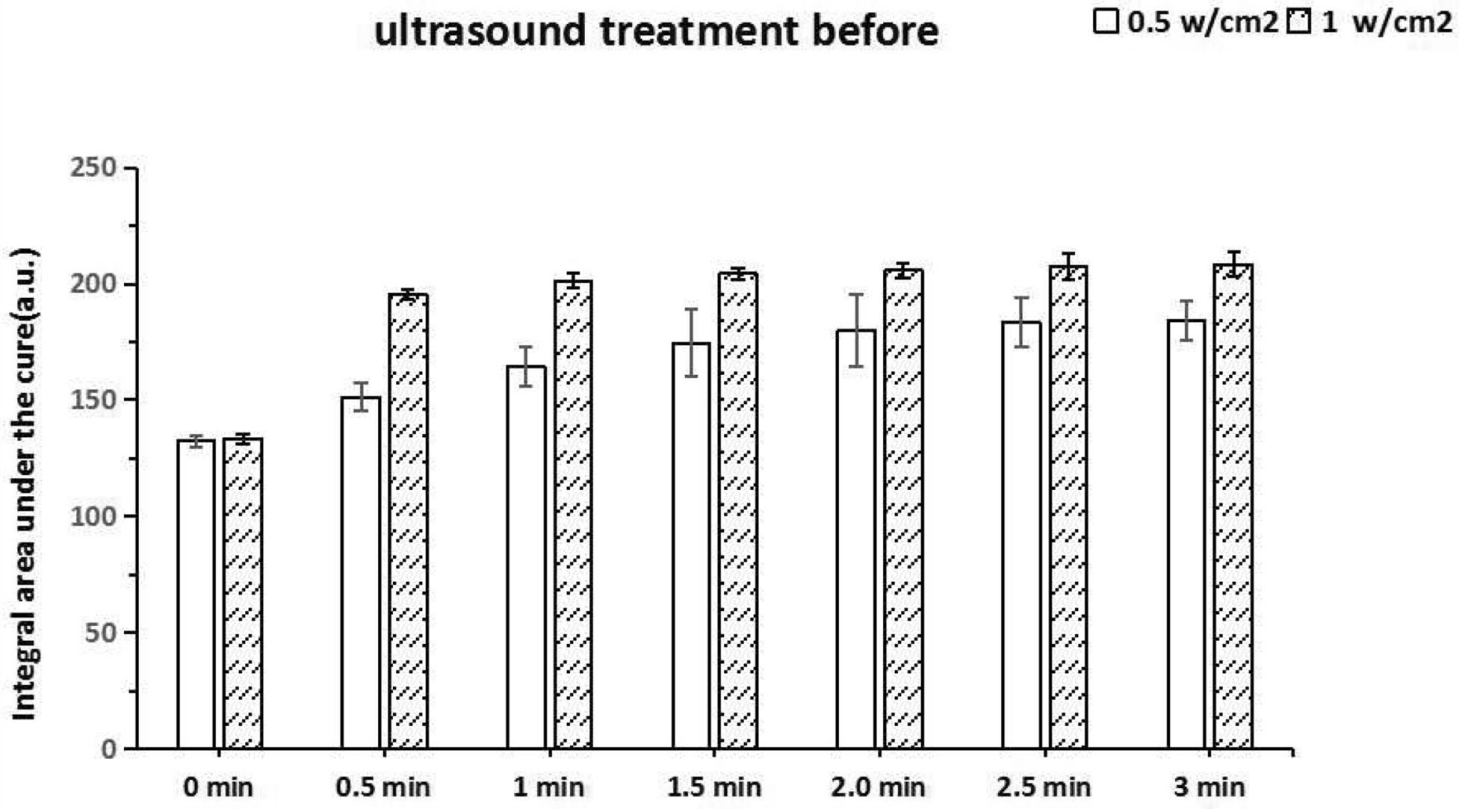
A comparative study on the integral area of HMME fluorescence spectrum curve of ultrasound treatment before group. The time from 0 to 3 min. Data represent mean values (*n* = 10), and error bars represent standard deviations. *Statistically significant differences between the two groups, *P* < 0.05

The penetration effect of ultrasound treatment before is shown in Fig. 7. After the corresponding intensity of sinusoidal ultrasound, adding 25 μg/mL of HMME 1 mL, the permeability of HMME has changed. There were statistical differences in each time point (*P* < 0.05). The change of permeability was positively correlated with the intensity of ultrasound.

**Fig. 8:**
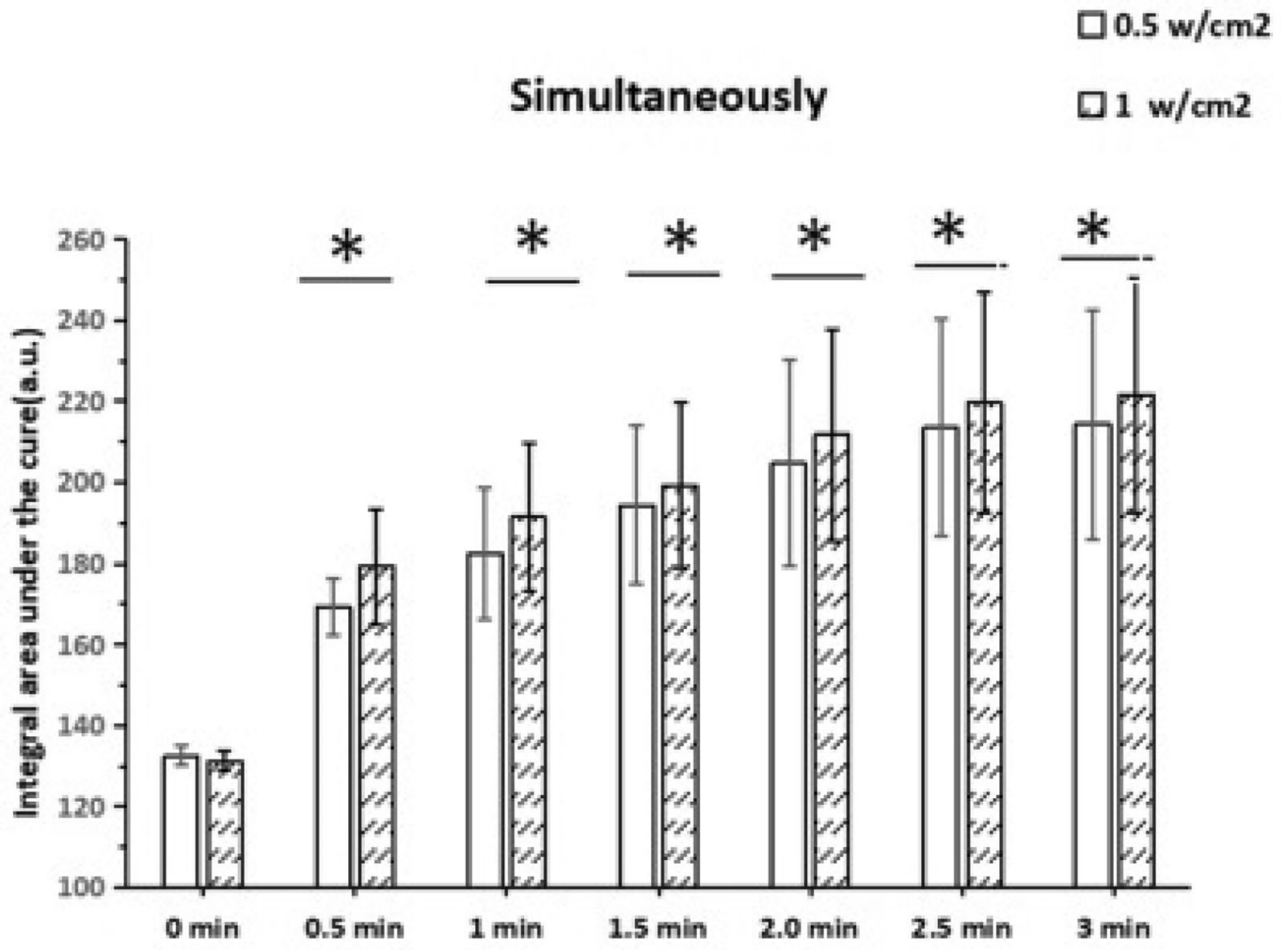
A comparative study on the integral area of HMME fluorescence spectrum curve of simultaneously group. The time from 0 to 3 min. Data represent mean values (*n* = 10), and error bars represent standard deviations. *Statistically significant differences between the two groups, *P* < 0.05

The penetration effect of simultaneously is shown in Fig. 8. Adding 25 μg/mL HMME 1 mL and at same time, adding sinusoidal ultrasound 0.5 and 1 W/cm^2^, respectively. The permeability of HMME has changed over the 6 time points (0.5, 1, 1.5, 2, 2.5 and 3 min) that were measured. There were statistical differences in each time point (*P* < 0.05), where the change of permeability was positively correlated with the intensity of ultrasound.

**Fig. 9:**
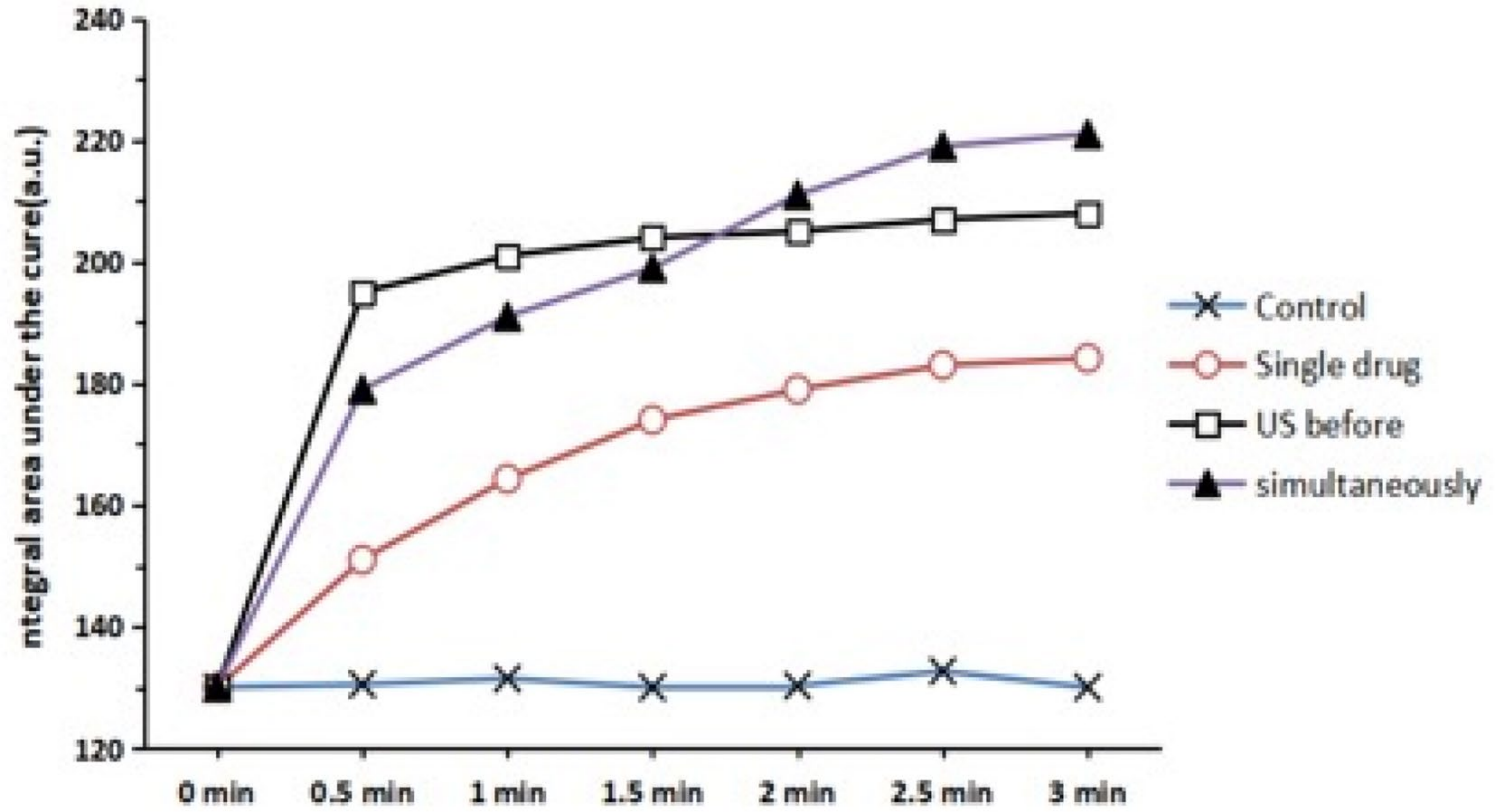
The effect of variation of HMME penetration in different action groups over time ranged from 0 to 3 min, and filled fork represented control group, filled circles single-drug group, filled squares ultrasound treatment before group, filled triangles simultaneously group

The values of S at each time point of the four experimental groups (control, single drug, ultrasound treatment before, simultaneously) were statistically analyzed as shown in Fig. 9. In the experiment, the ultrasonic intensity is 1 W/cm^2^. As shown in Fig. 9, HMME penetration in single-drug group shows a flat trend. A lower platform value is reached later. This value is significantly lower than the ultrasound treatment before group (*P* < 0.05) and the simultaneously group (*P* < 0.05). The permeation of HMME is relatively high at first, and then gradually decreased to a stable phase. This indicates that the effect of ultrasound on bacterial biofilm can be sustained for a period of time, and the effect is reversible. The simultaneously group is also with high HMME permeability in the early time, but significantly lower than ultrasound treatment before group (*P* < 0.05). Eventually, it reaches a platform value significantly higher than that of other groups (*P* < 0.05). This suggests that the simultaneously group may be the best delivery method, which can achieve a higher effective concentration.

## Discussion

Biofilm provides protection for bacterial cells from drugs and chemicals [18, 19]. This resistance can be explained by matrix impenetrability, QS (quorum sensing) activation and negative effects of the internal environment of the biofilm on antimicrobials, etc. [11, 20, 21]. The aim of this study is to establish a biofilm of *S. aureus*. Starting from the impenetrability of matrix, referring to the general scope of parameter selection of therapeutic ultrasound that does no harm to human cells [22–25] and study the penetration effect of low-frequency and low-intensity ultrasound rely on morphological and fluorescence spectrometric detection methods. If ultrasound is used as a simple and effective method to open the biofilm barrier, combined with the sterilization effect of PDT and SDT, it will have a broad application prospect. Many studies have shown that ultrasound can both kill bacteria and promote bacterial growth [9, 10, 26–28]. This is determined by a number of factors, such as the frequency and intensity of the ultrasound, the type of bacteria, materials used for ultrasonic diffusion, the appearance of cavitation, and forms of bacterial plankton or biofilms [27]. The cavitation effect of ultrasound can sterilize because the high-intensity ultrasound can mechanically destroy the polymer material, thus producing sterilizing effect. However, low-intensity ultrasound can stimulate the growth of bacteria and biofilm without sterilization. Studies on the destruction of biofilm also clearly show that ultrasound cannot kill bacteria at high intensity and high frequency, but it can cause damage to tissues [28–30].

Therefore, under the premise of maintaining the healthy tissue, low-frequency and low-intensity ultrasound was selected to study the effect of ultrasound on bacterial biofilm permeability, instead of damaging the integrity of biofilm. Seen from Fig. 5, the integrity of biofilm under macroscopic conditions can be guaranteed only when the intensity of sinusoidal ultrasound is less than 1 W/cm^2^. In some case, the data may be related to the way we culture bacteria and prepare biofilms. It has been reported that if this intensity frequency ultrasound is not combined with antibiotics, it is more likely to stimulate the growth of bacteria, presumably because the input of nutrients and oxygen [31–33]. Due to the complexity of bacterial biofilms, many mysteries remain to be solved. Many mechanisms related to low-frequency ultrasonic infiltration promotion have been proposed. The most basic and important mechanism is the mechanical effect [34]. Polat et al. [35] used low-frequency ultrasound and sodium dodecyl sulfate (SLS) to promote drug transdermal delivery. It is proved that the mechanical effect has a certain influence on improving the penetration ability of tissue. The essence of fluorescence analysis is that each substance emits a different fluorescence spectrum. Through the analysis of the spectrum, qualitative and quantitative analysis of the fluorescent substances can be made. Moreover, the sensitivity of this method is very high, which is hundreds of times higher than that of colorimetry or OD measurement [36]. The qualitative detection of fluorescent substances is mainly to observe whether the measured spectral curve has the characteristic wave peak. Quantitative analysis of fluorescent substances is mainly to compare the integral area under the spectral curve to determine the concentration of fluorescent substances [37]. As shown in Fig. 6, in a specific HMME concentration interval, the linear relationship between the integral area S and the corresponding solution concentration C exists: S = 10.335 C + 182.28. Therefore, it confirmed that the value of integral area S under the spectral curve can indirectly reflect the content of HMME in the *S. aureus* biofilm, and thus reflect the penetration of HMME in the biofilm.

In the low-frequency and low-intensity ultrasonic penetration promotion experiment, the relative HMME permeability can be compared and analyzed by measuring the integral area S under the curve of the fluorescence spectrum with different groups, different ultrasonic parameters and different time. As shown in Figs. 7 and 8, no matter whether it is ultrasound treatment before group or simultaneously group, the effect of promoting permeability with ultrasonic intensity of 1 W/cm^2^ is better than that with ultrasonic intensity of 0.5 W/cm^2^ at the same time point. In ultrasound treatment before group, through fluorescence spectrum analysis, it can be known that the permeability of biofilm increases significantly at the initial stage. This may because the pore size of the biofilm becomes larger or even the internal structure is damaged, so that the permeability of HMME increases [38].

However, this change does not persist. After a period of time, the change gradually disappears and returns to the pre-ultrasound level. In simultaneously group, with different ultrasonic intensities, there were significant differences in the integral area S value under HMME fluorescence spectrum curve of each group at each time (*P* < 0.05). The results indicated that the permeability of HMME to biofilm is significantly enhanced by low-frequency and low-intensity ultrasound. Moreover, the effect of ultrasound was positively correlated with the intensity and time of ultrasound when compared to three other groups; as shown in Fig. 9, under the same time, the integral area S value under the spectral curve of the simultaneous group significantly increased. This indicates that simultaneous action of ultrasonic and drugs may be the most reliable clinical application method, which is consistent with reports of other scholars [39].

Many infections are in the form of biofilms. Antimicrobial resistance increases exponentially once plaque biofilms are formed. Bacteria of different species in biofilms can live together harmoniously and produce a synergistic effect. Therefore, the use of low-frequency and low-intensity ultrasound to destroy the barrier effect of biofilm is of great significance for the clinical treatment of infectious diseases caused by biofilm. Low-frequency and low-intensity ultrasound can increase the penetration effect of drugs, break the barrier effect of biofilm and induce drugs to enter the biofilm [40, 41]. If PDT and SDT can be recombined, the prospect should be optimistic, but there is still a lot of work need to be done in the study of bacterial biofilms.

## Conclusions

Low-frequency and low-intensity sinusoidal ultrasound significantly increased the permeability of the biofilms, which was positively correlated with the time and the intensity of ultrasound. Simultaneous action of ultrasound and HMME was the most effective way to increase the permeability of the biofilms. This experiment only studied the biofilm formed by single bacteria of *Staphylococcus aureus*. Although *Staphylococcus aureus *is the most common bacteria causing infection, many clinical infections are caused by the biofilm formed by mixed bacteria, so it is still necessary to study the biofilm formed by mixed bacteria. According to the reports so far, the mechanism of ultrasonic action on bacteria and biofilms is still unclear. One of the key studies that needs to be done in the future is how can we decode bacterial quorum sensing and gene expression. A further question will be how to determine the mechanism of bacterial inactivation. Proteomics and other possible factors such as pressure, temperature, and chemical activity can be studied.

## Data Availability

All of the data and materials are available.
